# Reference values and biological determinants for cardiac myosin-binding protein C concentrations assessed with an enzyme-linked immunosorbent assay

**DOI:** 10.5937/jomb0-39081

**Published:** 2023-10-27

**Authors:** Sylwester M. Kloska, Marek Kozinski, Anna Stefanska, Katarzyna Bergmann, Aneta Mankowska-Cyl, Joanna Siodmiak, Grazyna Sypniewska, Magdalena Krintus

**Affiliations:** 1 Nicolaus Copernicus University, Collegium Medicum, Department of Forensic Medicine, Bydgoszcz, Poland; 2 Medical University of Gda sk, Department of Cardiology and Internal Medicine, Gdynia, Poland; 3 Nicolaus Copernicus University, Collegium Medicum, Department of Laboratory Medicine, Bydgoszcz, Poland

**Keywords:** biomarker, cardiac myosin-binding protein C, cMyBPC, cMyC, reference values, biomarker, srčani protein C koji se vezuje za miozin, cMiBPC, cMiC, referentne vrednosti

## Abstract

**Background:**

Cardiac myosin-binding protein C (cMyC) is a novel cardio-specific biomarker of potential diagnostic and prognostic value for cardiovascular events. This study aims to determine reference values for cMyC and identify biological determinants of its concentration.

**Methods:**

A population of 488 presumably healthy adults were enrolled to define biological determinants which affect cMyC concentrations in serum. Concentrations of cMyC were assessed using enzyme-linked immunosorbent assays from commercially available kits. Eligibility for inclusion in this study evaluated all subjects' anthropometric, demographic and laboratory measurements. After applying strict inclusion criteria, a reference population (n=150) was defined and used to determine reference values. Reference values were derived using a robust method.

## Abstract (sr)

**Uvod:**

Protein C koji vezuje srčani miozin (cMyC) je novi kardio-specifični biomarker koji ima potencijalnu dijagnostičku i prognostičku vrednost za kardiovaskularne događaje. Ova studija ima za cilj da odredi referentne vrednosti za cMyC i identifikuje biološke determinante njegove koncentracije.

**Metode:**

Da bi se definisale biološke determinante koje utiču na koncentracije cMyC u serumu, u studiju je uključeno 488 odraslih osoba za koje se pretpostavljalo da su zdrave. Koncentracije cMyC su procenjene korišćenjem enzimski vezanih imunosorbentnih testova iz komercijalno dostupnih kompleta. Podobnost za uključivanje u ovu studiju je procenjena antropometrijskim, demografskim i laboratorijskim merenjima kod svih ispitanika. Nakon primene strogih kriterijuma za uključivanje u studiju, definisana je referentna populacija (n=150) koja je korišćena za određivanje referentnih vrednosti. Referentne vrednosti su izvedene korišćenjem robusne metode.

**Rezultati:**

Distribucija koncentracija cMyC u referentnoj populaciji je bila neparametrijska i desno zakrivljena, sa samo 2 subjekta koji su imali koncentracije niže od granice detekcije. Ženski pol je bio jedina nezavisna determinanta viših koncentracija cMyC u verovatno zdravoj populaciji. Nije bilo značajne veze između cMyC i drugih ispitivanih parametara u referentnoj populaciji. Ukupna gornja referentna granica (URL) postavljena na 99. percentil za koncentraciju cMyC je bila 42,29 ng/mL i nije se razlikovala između žena i muškaraca (42,52 prema 42,35 ng/mL).

**Zaključak:**

Ova studija je uspe{no utvrdila referentne vrednosti za procenjeni cMyC test i istražila njegove biološke determinante. Uprkos uticaju ženskog pola na koncentracije cMyC u verovatno zdravoj populaciji, nismo otkrili polno zavisne razlike u cMyC na URL-u na 99. percentilu i stoga preporučujemo korišćenje jednog 99. percentilnog URL-a specifičnog za metodu za odrasle.

## Introduction

There is a clear need for the identification and clinical application of a reliable laboratory biomarker for myocardial infarction (MI), and of high diagnostic value in the early recognition of MI, particularly in patients with ambiguous symptoms and/or electrocardiographic findings.

Current clinical guidelines emphasise the importance of biomarkers of myocardial injuries, such as cardiac troponin (cTn), which increased blood concentration with at least one value above the 99th percentile upper reference limit (URL) being a diagnostic criterion for MI [1]. Unfortunately, measurements of cTn, even employing high-sensitivity (hs) assays, may fail to detect MI in early presenters, with its mildly elevated results lacking specificity in MI diagnosis.

Cardiac myosin-binding protein C (cMyC) is considered a candidate biomarker to facilitate the early diagnosis of MI. Discovered in 1973, myosinbinding protein C is a core protein that controls or modifies muscular cross-bridge movements and stabilises the filaments [2] [3]. There are three isoforms of myosin-binding protein C in human muscles, i.e. fast and slow skeletal muscle isoforms, encoded by MYBPC1 and MYBPC2 genes, respectively, and cardiac isoform (cMyC), encoded by the MYBPC3 gene. Being part of the thick filament of cardiomyocytes, cMyC restrains interactions between myosin and actin by combining with the rod region of myosin [4]. The biological activity of cMyC is closely related to its phosphorylation which is essential in proper myocardial function and with potentially protective facility for ischemic injury.

Due to the important physiological role of cMyC in maintaining normal contractions of heart muscle fibres, this protein is constantly present in the blood [5]. In severe myocardial ischemia, its N-terminal part undergoes proteolysis and cMyC is released into the bloodstream in relatively high amounts, reaching levels even 2-fold higher than those observed for cTn [6]. This feature may potentially aid the distinction between elevated and physiological cMyC concentrations hence increasing the usefulness of this biomarker in MI detection. A study in rats showed a substantial increase in cMyC plasma concentrations within 30 minutes of acute myocardial injury [7]. Another advantage of cMyC is the shorter clearance time (12 hours as opposed to 10–14 days for cTn) which may enable the diagnosis of recurrent MI.

To enhance our understanding of physiological cMyC concentrations and to facilitate its clinical application, this study aimed to determine a URL for cMyC concentrations and to identify biological determinants of cMyC concentrations in healthy subjects.

## Materials and methods

### Study conduction

**Figure 1:**
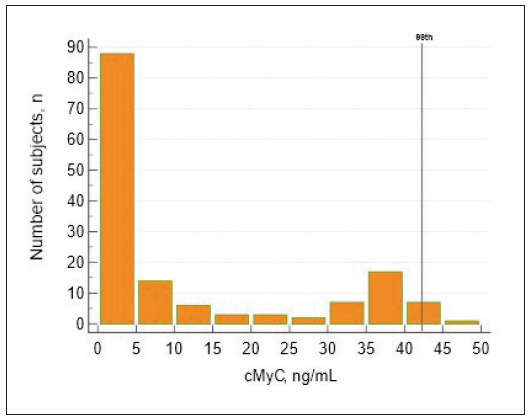
Distribution of cMyC concentrations in the reference population (n=150) The continuous line indicates the 99^th^ URL (42.29 ng/mL).<br>cMyC, cardiac myosin-binding protein C

The study was performed in 150 clinically healthy subjects, designated the reference population and drawn from a larger, presumably healthy cohort of 488 individuals who were previously sampled for our studies on reference values for hs-cTnI [8], galectin-3 [9], mid-regional pro-adrenomedullin [10] and growth differentiation factor 15 [11]. The study participants were Caucasians recruited from two Polish cities (Bydgoszcz and Torun). Blood samples were sourced twice, in 2013 and again in 2015. Enrolled subjects had no known active or chronic inflammatory disease at the time of collection and were not undergoing treatment with immunosuppressive agents, non-steroidal anti-inflammatory drugs, steroids, or antibiotics, and none were pregnant. Before the blood draw, all individuals gave informed written consent to participate in the study and answered a questionnaire based on which an initial screening was undertaken, excluding 65 individuals with either hypertension, diabetes, or both. One participant did not answer appropriate questions. The remaining 422 subjects (aged 19–84 years) were classified as the 'presumably healthy' population. Further screening of this group allowed us to define a clinically healthy reference population to determine cMyC reference values. Parameters for inclusion in the reference population were based on the results of laboratory tests within the following limits: hs-cTnI <16 ng/L in females and <34 ng/L in males [12], B-type natriuretic peptide (BNP) <35 ng/L [13], C-reactive protein (CRP) <10 mg/L [14], glycated hemoglobin (HbA_1c_) <42 mmol/mol [15], and an estimated glomerular filtration rate (eGFR), employing the Chronic Kidney Disease-Epidemiology Collaboration equation >90 mL/min/1.73 m^2^ [16]. Other criteria for inclusion in the reference group were body mass index (BMI) <30 kg/m^2^ and serum concentrations of total cholesterol (TC) <6.22 mmol/L, and triglycerides (TG) <2.26 mmol/L. Subjects not meeting at least one of these criteria were excluded from the reference group. Following this robust protocol, a well-defined reference population of 150 physiologically and clinically healthy individuals (aged 19–62) was selected to determine reference values for cMyC (Figure 1).

### Laboratory measurements

In the studies mentioned above [8] [9] [10] [11], all laboratory tests, except for cMyC, were undertaken on fresh blood samples. Serum was obtained within less than an hour to avoid proteolysis and stored deepfrozen (-80°C) in small aliquots until assayed for cMyC concentration. Concentrations of BNP, HbA_1c_, creatinine, basic lipid profile and hs-cTnI were measured on the Abbott Architect ci8200 analyser using commercially available assays (Abbott Laboratories, Wiesbaden, Germany). CRP measurements were performed on the Horiba ABX Pentra 400 analyser (Horiba ABX, Montpellier, France).

Concentrations of cMyC were determined using enzyme-linked immunosorbent assay (ELISA) from a commercially available kit (SunRed Biotechnology Co., Ltd., Shanghai, China). The assay procedure was performed under the manufacturer's instructions. The limit of detection (LoD) of this assay was 0.122 ng/mL, and the assay range was 0.15–32 ng/mL. The coefficient of variation (CV) reported by the manufacturer was less than 10%. No significant crossreactivity between human cMyC and analogues was observed.

### Statistical methods

The Shapiro-Wilk test was used to determine normality in the distribution of variables in the study group. The Mann-Whitney U-test was used to evaluate significant differences between the two groups. Differences among qualitative variables were compared with a chi-square test. Relationships between concentrations of cMyC and other investigated parameters were determined using the Spearman correlation coefficient. The influence of potential determinants on the variability of serum concentrations of cMyC was assessed through multivariate regression analysis. Results were considered statistically significant if p<0.05. A URL was designated at the 99 percentile and calculated using a robust method recommended in CLSI EP28-A3c [17]. Outliers were detected according to Reed's method [18]. All statistical analyses were performed using MedCalc version 20.011 (MedCalc Software Ltd, Ostend, Belgium).

## Results

### Characteristics of study populations

Table 1 presents baseline characteristics for the presumably healthy population and the reference group. Gender and smoker distributions were similar in both groups. Participants in the reference group were markedly younger and had lower BMI than in the presumably healthy population. Additionally, statistically significant lower concentrations of BNP, TC, low-density lipoprotein cholesterol (LDL-C), non-high-density lipoprotein cholesterol (non-HDL-C), TG, CRP and higher eGFR values were observed in the reference population. A median concentration of cMyC was numerically lower in presumably healthy subjects than in the reference group. Inversely was observed for hs-cTnI, i.e. with non-significantly higher values in presumably healthy subjects. Comparison of female and male groups showed statistically higher BMI, LDL-C, non-HD-C, TG and eGFR values and a lower level of HDL-C in males from the presumably healthy and reference populations.

**Table 1:** Characteristics of the study participants $ p<0.05, & p<0.01*<0.001; # p<0.0001 A vs B or C vs D or E vs F. Quantitative variables are expressed as medians, and 1st–3rd quartile ranges and categorical data as numbers and percentages. BMI, body mass index; BNP, B-type natriuretic peptide; cMyC, cardiac myosin-binding protein C; CRP, C-reactive protein; eGFR, estimated glomerular filtration rate; HbA1c, glycated hemoglobin; hs-cTnI, high-sensitivity cardiac troponin I; HDL-C, high-density lipoprotein cholesterol, LDL-C, low-density lipoprotein cholesterol; TC, total cholesterol; TG, triglycerides.

Variable	Presumably<br>healthy<br>population<br> (n=422) (A)	Reference<br>population<br>(n=150)<br>(B)	Presumably<br>healthy<br>females<br>(n=223) (C)	Presumably<br>healthy<br>males<br>(n=199) (D)	Reference<br>population<br>females<br>(n=78) (E)	Reference<br>population<br>males<br>(n=72) (F)
Age, years	40<br>(33–52)	35.5<br>(30–43) #	40<br>(34–53)	40<br>(32–51)	35<br>(30–39)	39<br>(31–47) $
Gender, females	223<br> (53%)	78<br>(52%)	–	–	–	–
BMI, kg/m^2^	25.1<br>(22.5–28.1)	23.7<br>(21.6–26.2) #	23.8<br>(21.3–27.1)	26.6<br>(24.2–29.4) #	22.0<br>(20.4–24.2)	25.0<br>(23.5–27.4) #
cMyC, ng/mL	2.61<br>(1.18–15.73)	3.15<br>(1.39–19.0)	3.91<br>(1.33–26.73)	2.01<br>(0.98–8.93) *	4.69<br>(1.64–24.04)	2.47<br>(1.10–13.72)
hs-cTnI, ng/L	2.45<br>(1.7–3.3)	2.3<br>(1.7–3.1)	2.40<br>(1.70–3.0)	2.60<br>(1.70–3.80) $	2.20<br>(1.80–2.90)	2.50<br>(1.50–3.40)
BNP, ng/L	15.0<br>(10.0–23.9)	13.35<br>(10.0–16.8) *	17.7<br>(10.5–28.9)	11.4<br>(10.0–18.8) #	14.5<br>(10.0–17.2)	10.7<br>(10.0–16.4)
HbA_1c_,<br>mmol/mol	35.5<br>(32.2–38.8)	35.5<br>(32.2–37.7)	35.5<br>(33.0–38.0)	36.0<br>(32.0–38.8)	35.5<br>(32.2–36.6)	36.0<br>(31.5–38.8)
eGFR,mL<br>/min/1.73 m^2^	93<br>(86–103)	99<br>(94–107) #	90<br>(84–97)	97<br>(89–110) #	97<br>(94–103)	104<br>(94–112) &
TC, mmol/L	5.18<br>(4.53–6.00)	4.95<br>(4.34–5.38) *	5.18<br>(4.45–5.98)	5.23<br>(4.58–6.03)	4.92<br>(4.22–5.33)	4.97<br>(4.45–5.52)
HDL-C, mmol/L	1.45<br>(1.22–1.71)	1.47<br>(1.22–1.71)	1.58<br>(1.42–1.86)	1.24<br>(1.06–1.42) #	1.58<br>(1.42–1.89)	1.24<br>(1.13–1.48) #
LDL-C, mmol/L	3.13<br>(2.53–3.88)	2.84<br>(2.38–3.47) *	2.95<br>(2.38–3.76)	3.34<br>(2.72–4.04) &	2.69<br>(2.20–3.29)	3.08<br>(2.62–3.70) *
Non-HDL-C,<br>mmol/L	3.74<br>(3.00–4.53)	3.36<br>(2.77–3.98) #	3.47<br>(2.80–4.30)	4.01<br>(3.34–4.77) #	3.06<br>(2.64–3.68)	3.63<br>(3.11–4.27) #
TG, mmol/L	1.11<br>(0.82–1.72)	0.90<br>(0.70–1.21) #	0.95<br>(0.71–1.39)	1.40<br>(0.99–1.99) #	0.77<br>(0.62–1.02)	1.08<br>(0.87–1.53) #
CRP, mg/L	0.58<br>(0.22–1.81)	0.36<br>(0.13–0.70)	0.63<br>(0.19–2.29)	0.51<br>(0.22–1.42)	0.26<br>(0.12–0.69)	0.36<br>(0.16–0.74)
Current or<br>former smoker	151<br>(36%)	52<br>(35%)	71<br>(32%)	82<br>(42%) $	23<br>(30%)	29<br>(40%)

Eight subjects displayed cMyC concentrations below the LoD (<0.122 ng/mL), with 6 in the presumably healthy population and 2 in the reference group.

### Biological determinants influencing cMyC concentration

In the presumably healthy population, concentrations of cMyC were significantly higher in females than in males (Table 2). Conversely, differences between women and men in cMyC concentrations did not reach the significance level in the reference population (Table 2 and Figure 1). Moreover, there were no statistically significant differences in concentrations of cMyC dependent on age in the presumably healthy population or the reference group.

**Table 2:** cMyC concentrations in the presumably healthy population and in the reference group stratified by gender and age cMyC, cardiac myosin-binding protein C; IQR, interquartile range; N/A, not applicable. 75^th^ percentiles of cMyC in presumably healthy and reference populations were 15.73 ng/mL and 19.0 ng/mL, respectively.

Category	n	Median [ng/mL]	IQR [ng/mL]	Range [ng/mL]	p-value
Presumably healthy population (n=422)					
Overall	422	2.61	1.18–15.73	<0.12–47.73	N/A
<40 years	201	2.62	1.24–13.14	<0.12–47.47	0.751
40 years	221	2.37	1.18–22.89	<0.12–47.73	
Male	199	2.01	0.98–8.93	<0.12–46.02	0.0003
Female	223	3.91	1.33–26.73	<0.12–47.73	
Males <75^th^ percentile of cMyC	159	1.49	0.83–2.66	<0.12–15.41	0.004
Females <75^th^ percentile of cMyC	158	1.85	1.17–4.43	<0.12–15.72	
Males ≥75^th^ percentile of cMyC	40	37.28	27.57–40.41	16.44–46.00	0.413
Females ≥75^th^ percentile of cMyC	65	37.73	33.34–40.07	18.21–47.73	
Reference population (n=150)					
Overall	150	3.15	1.39–19.00	<0.12–46.02	N/A
<40 years	100	3.35	1.74–20.73	<0.12–46.02	0.114
40 years	50	1.88	1.05–14.78	<0.12–42.13	
Male	72	2.47	1.10–13.72	<0.12–46.02	0.069
Female	78	4.69	1.64–24.04	<0.12–40.88	
Males < 75^th^ percentile of cMyC	56	1.71	0.85–5.47	<0.12–15.41	0.021
Females <75^th^ percentile of cMyC	57	2.39	1.41–5.47	<0.12–19.00	
Males ≥75^th^ percentile of cMyC	16	38.56	34.93–41.28	23.16–46.02	0.122
Females ≥75^th^ percentile of cMyC	21	35.75	35.75–38.94	22.45–40.87	

Additionally, we calculated cMyC medians in subgroups of females and males with lower cMyC values (i.e. <75^th^ percentile) and higher cMyC values (≥75 th percentile). This analysis showed that the difference in cMyC concentrations between genders becomes insignificant in a higher concentration range of cMyC (Table 2).

The comparison of female subgroups with cMyC concentrations below and above the 75^th^ percentile of cMyC showed a tendency to higher concentrations of HbA_1c_ (p=0.065), TC (p=0.053), LDL-C (p=0.083) and non-HDL-C (p=0.102) in females with cMyC concentrations above or equal to 75^th^ percentile of cMyC. These tendencies were not observed during the analysis of male subgroups.

Table 3 shows Spearman correlation results. The correlation analysis indicated weak but statistically significant relationships between concentrations of cMyC and BMI (negative correlation), HDL-C (positive correlation) and triglycerides (negative correlation) in the presumably healthy population (n=422). We failed to find any associations between cMyC concentration and other investigated parameters in the reference population.

**Table 3:** Correlations between cMyC concentration and age, BMI and selected laboratory parameters in the presumably healthy population (n=422), and in the reference population (n=150) BMI, body mass index; BNP, B-type natriuretic peptide; cMyC, cardiac myosin-binding protein C; CRP, C-reactive protein; eGFR, estimated glomerular filtration rate; HbA1c, glycated hemoglobin; HP, healthy population; hs-cTnI, high-sensitivity cardiac troponin I; HDL-C, high-density lipoprotein cholesterol, LDL-C, lowdensity lipoprotein cholesterol; Rs, Spearman’s coefficient of rank correlation rho; TC, total cholesterol; TG, triglycerides.

Variable	Presumably healthy<br>population		Reference<br>population	
	R_s_	p-value	R_s_	p-value
Age	-0.020	0.677	-0.111	0.178
BMI	-0.148	0.003	-0.091	0.270
hs-cTnI	0.012	0.803	-0.057	0.487
BNP	0.098	0.059	0.027	0.748
HbA1c	0.055	0.262	0.043	0.602
eGFR	0.077	0.117	-0.0039	0.963
TC	0.015	0.761	-0.055	0.504
HDL-C	0.105	0.031	0.041	0.622
LDL-C	0.0110	0.823	-0.039	0.634
Non-HDL-C	-0.03	0.543	-0.073	0.378
TG	-0.158	0.001	-0.105	0.203
CRP	-0.073	0.138	-0.115	0.162

Additionally, we separately analysed Spearman correlations between cMyC levels and biological determinant values in the lower and higher concentration ranges of cMyC. We found weak negative correlations (all p<0.05) between cMyC concentration and age (Rs=-0.11), TG (R_s_=-0.18), CRP (Rs - 0.12), and the weak positive correlations with HDL-C (Rs=0.15) and eGFR (Rs=0.13) in the subgroup of the presumably healthy population with a lower concentration of cMyC (<75^th^ percentile; n=317). In contrast, the opposite trend in correlations was observed in the subgroup of the presumably healthy population characterised by higher concentrations of cMyC (≥75^th^ percentile, n=105): age (R_s_=0.20), TG (Rs=0.12), CRP (Rs=0.13). Interestingly, the strongest positive relationships (all p<0.05) between cMyC and biological determinants were observed in the subgroup of presumably healthy females with higher cMyC values (≥75th percentile; n=65): HbA1c (R_s_=0.26), non-HDL-C (R_s_=0.28), LDL-C (R_s_=0.26) and TG (R_s_=0.28).

A multiple regression model analysis (n=422) was undertaken to define factors influencing the concentration of cMyC in the presumably healthy population (Table 4) and the reference population (Table 1). We identified the female gender as the only independent determinant of higher cMyC concentrations in the presumably healthy population. However, all these models poorly explained the variability of cMyC concentrations. None of the investigated variables was associated with cMyC concentrations in the reference population.

**Table 4:** Impact of selected variables on cMyC concentrations in multiple regression analysis in the presumably healthy population (n=422) BMI, body mass index; cMyC, cardiac myosin-binding protein C; HDL-C, high-density lipoprotein cholesterol.

Regression models	Model characteristics
Model adjusted for gender and BMI	R^2^=0.0285<br>Significant determinant of higher cMyC concentrations:<br> female gender (p=0.014).<br> Lack of impact: BMI.
Model adjusted for gender, BMI, and smoking	R^2^=0.0303<br> Significant determinant of higher cMyC concentrations:<br>female gender (p=0.017).<br>Lack of impact: BMI and smoking.
Model adjusted for gender, BMI, HDL-C,<br>and triglycerides	R^2^=0.03<br>Significant determinant of higher cMyC concentrations:<br>female gender (p=0.013).<br>Lack of impact: BMI, HDL-C, and triglycerides.
Model adjusted for gender, BMI, HDL-C, triglycerides,<br>and smoking	R^2^=0.0318Significant determinant of higher cMyCconcentrations: female gender (p=0.013).Lack of impact: BMI, HDL-C, triglycerides, and smoking.

### Reference values for cMyC concentrations in serum

The distribution of cMyC concentrations in the reference population was non-parametric and rightskewed, as presented in Figure 1. The derived 99^th^ URL for cMyC concentration was 42.29 ng/mL. The 99^th^ percentile URL was 42.52 for women and 42.35 ng/mL for men.

## Discussion

As far as we are aware, this is one of the very few studies to date on reference values for cMyC [19]. We derived a 99^th^ URL for this biomarker in a healthy population after applying a protocol based on stringent selection criteria, as we had done previously for other biomarkers [8] [9] [10] [11]. Interestingly, we demonstrated that the female gender contributed significantly to higher cMyC concentrations in the presumably healthy population but not in the healthy cohort. Furthermore, the derived URLs did not differ between women and men. This study's findings are important in understanding physiological cMyC concentrations and allow for the further evaluation of its clinical usefulness.

The diagnosis of MI based on the assessment of biomarkers of myocardial injury is still a matter of much controversy among clinicians. According to recent guidelines of the European Society of Cardiology and the 4^th^ Universal Definition of Myocardial Infarction, a mandatory condition for diagnosing MI is the detection of the rise and/or fall of cardiac biomarkers, primarily cTn [1] [20]. The introduction of highly sensitive tests for cTn significantly increased the percentage of »troponin-positive« patients, though not necessarily »MI-positive« cases during the acute ischemic event. Due to the non-specific release of cTn from damaged myocardium, extending hs-cTnI testing as much as 3–6 hours after chest pain onset is sometimes recommended. During this period, many patients are at an undefined risk with too high cTn concentrations for discharge but too low to be diagnosed with MI [21]. Therefore, searching for new, early, cardiac-specific biomarkers is extremely important to improve the efficacy of triage and MI diagnosis.

Determining reference values for candidate biomarkers is a necessary step in their further research leading to the validation of their clinical utility. In our study, we established a 99^th^ percentile URL for cMyC concentrations in serum and evaluated whether its concentration is associated with lifestyle factors such as smoking or other laboratory parameters. Because cMyC is a potential diagnostic and prognostic marker for cardiovascular events, it should be considered alongside other markers, i.e. cardiac troponins. Studies on the variability of cTn concentrations in the population indicate higher cTn levels in older adults [22] [23] [24]. Although, our study failed tofind any impact of age on cMyC concentrations either in the presumably healthy population or in the clinically healthy reference population. Tong et al. [25] demonstrated that significant amounts of cMyC have been observed in individuals, irrespective of ischemic injury and age.

We expected that similarly to cTn [12] [23] [24] [26], cMyC concentrations would be higher in males, which differences in the size and weight of the heart muscle might explain. However, our study indicates that higher concentrations of cMyC were found in females in the presumably healthy population. Nevertheless, in the reference group, the difference in cMyC values between females and males was not more statistically significant (p=0.069). Accordingly, we did not observe the difference in reference values between genders. Our observations support the lack of difference in URLs between females and males that cMyC levels were not statistically different between females and males in a higher concentration range of cMyC (≥75^th^ percentile). While the concentrations of cMyC were significantly higher in females in a lower concentration range of cMyC (<75^th ^percentile). To explain this phenomenon, we separately analysed associations between biological determinants and cMyC levels in males and females. We found that women had a more favourable cardiometa bolic profile when compared to men. Additionally, we found that the presumably healthy women with cMyC levels <15.73 ng/mL (75th percentile) tended to lower HbA1c, TC, LDL-C and non-HDL-C concentrations compared to women with cMyC levels ≥15.73 ng/mL. Moreover, the correlation analyses showed that cMyC level correlated negatively with cardiometabolic risk factors in the subgroup with a lower concentration range of cMyC. While in a higher concentration range of cMyC, positive trends in these correlations were observed, especially in females. Taken together our findings, we could hypothesise that higher serum concentrations of cMyC reflect a more favourable risk profile in females with serum cMyC concentration in a lower range (levels at least 2.7 times lower than URL value). It is well-known that phosphorylated cMyC has a protective function in heart tissue (enhances diastolic function, mediates inotropy, and confers heart protection during ischemia) [25]. As was mentioned above, circulating serum levels of cMyC are observed in also in healthy individuals, irrespective of the ischemic injury [25]. This suggests that cMyC concentrations observed in a presumably healthy population are regulated by physiological factors, including gender and sex hormones [27]. Thus, we cannot exclude the hypothesis that higher serum levels of cMyC may reflect a protective function of this protein in females in a health condition. Also of note is the potential inter-relationship between concentrations of cTn and those of cMyC. Due to the damage of cardiomyocytes, particularly in the course of MI [28], these proteins may enter the bloodstream, suggesting that their concentrations may be correlated. Rather surprisingly, our results did not show this relationship in the presumably healthy or reference populations. In contrast, Kaier et al. [26] found a significant elevation of cMyC concentrations in patients diagnosed with MI. The diagnostic value of cMyC, determined by receiver operating characteristic (ROC)-curves (area under the curve [AUC]=0.924), was comparable to hs-cTnT (AUC=0.927) and hs-cnTnI (AUC=0.922), and superior to cTnI when measured with contemporary assay (AUC=0.909). Moreover, in early presenters (with elapsed time between the onset of chest pain and blood sampling <3 hours), cMyC improved the rule in/rule out classification compared to both hs-cTnI and hs-TnT. In another study [29], Kaier et al. [26] evaluated the correlation between concentrations of cMyC and hs-cTnI in patients diagnosed with type 1 MI. The relationship was classified as significant upon admission, after 3 hours and at the late time point. The cMyC/hs-cTnI concentration ratio was highest upon admission and decreased in successive time intervals. These results indicate that cMyC may be an effective biomarker as its concentration in the blood increases very quickly enabling faster diagnosis. In particular, a combination of both biomarkers in creased the accuracy of correct diagnosis in suspected MI patients. However, there is no evidence to date to suggest that serum concentrations of cMyC and cTnI are correlated in healthy individuals. The differences between cTn and cMyC may be explained by different locations on cardiomyocytes, and the abundance and susceptibility to proteolysis or phosphorylation, which finally contribute to the release and degradation of cardiac proteins in circulation [30]. Detectable cMyC concentrations in healthy subjects may reflect normal cell turnover and protect against myocardial ischemia [25].

cMyC concentrations in healthy individuals are poorly documented, and robust reference values are still lacking. However, it should be noted that none of the immunochemistry methods for cardiac markers measurements is currently standardised or even harmonised despite many actions undertaken by experts [31]. Due to this, immunoassays are highly dependent on the type and quality of the antibodies used. Moreover, assay specificity is influenced by the heterogeneity of epitopes in the proteins (intact-full length and fragmented parts of cardiac biomarkers in circulation, e.g. cMyC appears in the serum as fulllength, approx. 140 kDa and fragmented protein, approx. 40 kDa [6]. Therefore, the results obtained by using assays with different epitope recognition by antibodies may give different results. In addition, inappropriate calibrator epitopes may cause inaccurate results, especially when a calibrator used is not identical concerning epitopes targeted by the assay (recombinant vs blood purified proteins) [32]. Another problem is using immunological methods with different final signal detection. The use of electrochemiluminescence for signal detection is more sensitive than a colourimetric reaction, which is used in ELISA methods [30].

All these aspects are responsible for discrepancies between methods. This emphasised that medians and reference values should be interpreted as method-specific. The assessment of reference values should also consider the study participants' selection criteria. Our median values in presumably healthy and reference populations were 2.61 and 3.15 ng/mL, respectively, with a URL value of 42.29 ng/mL. Govin dan et al. [33] observed higher values in control individuals, 22.3±2.4 ng/mL. According to the manufacturer's specifications, this MYBPC3 ELISA Kit (Human) from Aviva Systems Biology has a Detection Range of 0.156–10 ng/mL [34]. According to the Biovednor assay for human MyBPC3 ELISA, among unselected healthy donors (n=70), 68 samples were measured under the lowest standard of 10 pg/mL and 2 samples were measured between 10 and 20 pg/mL [35].

A paper worthy of discussion by Alaour et al. [36] employed an electrochemiluminescence method (Erenna® platform by EMD Millipore Corporation), and results differed significantly from those obtained in our study (with median concentrations of 4.38 vs 3.15 ng/L, respectively). Another explanation could be a completely different approach to the study, as the purpose of Alour et al. was to determine the individual biological variability of cMyC. In addition, both studies involved a widely different number of participants (150 in our reference population vs 30 in Alaour's) and various selection criteria. Inclusion criteria for [36] study were less stringent as they included individuals with eGFR>60 [mL/min/1.73 m^2^], while in our population, it was eGFR>90 [mL/min/1.73 m^2^]. Due to these differences, we believe that obtained results of both studies should not be directly compared.

Some limitations of our study should be acknowledged. Firstly, the study population was medium-sized and exclusively Caucasian. Although Hickman et al. [37] suggested that the sample size required to determine the 99th population percentile should include at least 300 healthy individuals and ideally 500, our study protocol was in line with CLSI guidelines on reference values [17]. Further, serum samples in our study were stored at -80°C before measurement, although protein markers are known to be sufficiently stable to withstand prolonged storage and produce viable results. Additionally, we used the traditional ELISA assay, a readily available, cost-effective and widely used research tool, although it is less sensitive and lacks the precision of automated assays. Unfortunately to date, there has only been one electrochemiluminescence assay developed for cMyC measurements on the Erenna analytical platform. Finally, methodological differences in the application of assays and the relative quality of antibodies make comparison difficult between methods, limitations which are especially noticeable when using this biomarker.

## Conclusions

In conclusion, we successfully established reference values for the assessed cMyC assay and investigated their biological determinants. Despite the impact of female gender on cMyC concentrations in the presumably healthy population, we did not detect sex-dependent differences in the cMyC 99th URL, and we recommend using a single method-specific 99th URL for adults.

## Dodatak

### Acknowledgements

This study was supported by the Collegium Medicum of Nicolaus Copernicus University (NCU CM grant no. 779/2014). Abbott Laboratories partially provided the reagents, calibrators and controls for this study.

### Conflict of interest statement

All the authors declare that they have no conflict of interest in this work.

### List of abbreviations

AUC, the area under the curve; 

BMI, body mass index; 

BNP, B-type natriuretic peptide; 

cMyC, cardiac myosin-binding protein C; 

CRP, C-reactive protein; 

cTn, cardiac troponin; 

CV, coefficient of variation; 

eGFR, estimated glomerular filtration rate; 

ELISA, enzyme-linked immunosorbent assay; 

HbA1c, glycated hemoglobin; 

HF, heart failure; 

hs-cTnI, high-sensitivity cardiac troponin I; 

HDL-C, high-density lipoprotein cholesterol, 

IQR, interquartile range; 

LDL-C, low-density lipoprotein cholesterol; 

MI, myocardial infarction; 

N/A, not applicable; 

ROC, receiver operating characteristic; 

RS, Spearman’s coefficient of rank correlation rho; 

TC, total cholesterol; 

TG, triglycerides; 

URL, upper reference limit.
